# Fully endoscopic microvascular decompression for hemifacial spasm: a clinical study and analysis

**DOI:** 10.1007/s10143-024-02311-5

**Published:** 2024-02-16

**Authors:** Xialin Zheng, Binbin Zhang, Dongqi Shao, Longjie Cai, Shan Xie, Yu Li, Zhiquan Jiang

**Affiliations:** 1 Department of Neurosurgery, The First Affiliated Hospital of Bengbu Medical University, Bengbu, China; 2 School of Continuing Education, Anhui Medical University, Hefei, China

**Keywords:** Neuroendoscopy, Facial spasm, Microvascular decompression, Endoscope

## Abstract

Fully endoscopic microvascular decompression (MVD) of the facial nerve is the main surgical treatment for hemifacial spasm. However, the technique presents distinct surgical challenges. We retrospectively analyzed prior cases to consolidate surgical insights and assess clinical outcomes. Clinical data from 16 patients with facial nerve spasms treated at the Department of Neurosurgery in the First Affiliated Hospital of Bengbu Medical College, between August 2020 and July 2023, were retrospectively examined. Preoperatively, all patients underwent magnetic resonance angiography to detect any offending blood vessels; ascertain the relationship between offending vessels, facial nerves, and the brainstem; and detect any cerebellopontine angle lesions. Surgery involved endoscopic MVD of the facial nerve using a mini Sigmoid sinus posterior approach. Various operative nuances were summarized and analyzed, and clinical efficacy, including postoperative complications and the extent of relief from facial paralysis, was evaluated. Fully endoscopic MVD was completed in all patients, with the offending vessels identified and adequately padded during surgery. The offending vessels were anterior inferior cerebellar artery in 12 cases (75%), vertebral artery in 3 cases (18.75%), and posterior inferior cerebellar artery in 1 case (6.25%). Intraoperative electrophysiological monitoring revealed that the lateral spread response of the facial nerve vanished in 15 cases and remained unchanged in 1 case. Postoperative facial spasms were promptly alleviated in 15 cases (93.75%) and delayed in 1 case (6.25%). Two cases of postoperative complications were recorded—one intracranial infection and one case of tinnitus—both were resolved or mitigated with treatment. All patients were subject to follow-up, with no instances of recurrence or mortality. Fully endoscopic MVD of the facial nerve is safe and effective. Proficiency in endoscopy and surgical skills are vital for performing this procedure.

## Introduction

Hemifacial spasm (HFS) is a condition caused by facial nerve dysfunction and is characterized by involuntary, paroxysmal twitch of facial muscles. When the patient is excited or nervous, the condition may be aggravated. Severe cases can experience a crooked mouth and difficulty opening the eyes. Although HFS is not life-threatening, it causes serious psychological burden and directly affects the quality of life of patients. The global annual incidence of HFS is approximately 11 cases/100,000 people. HFS does not respond well to drug treatment [1]. Thus, microvascular decompression (MVD) is the most effective approach for treating HFS [2, 3].

Historically, surgeons employed microscopes for MVD surgeries utilizing the Sigmoid sinus posterior approach. However, rapid advancement of neuroendoscopic technology means there is potential for surgeons to leverage the benefits of neuroendoscopy, including a clear field of view, multi-angle perspectives, and close observation capabilities, when conducting entirely endoscopic MVD of the facial nerve. In 2021, Zhao [4] compared and analyzed microsurgical and neuroendoscopic MVD in the treatment of Chinese patients with HFS, with 1122 patients (microsurgical MVD: 562 patients; neuroendoscopic MVD: 560 patients) included in the meta-analysis. The results of the study suggest that although microsurgical MVD is relatively simple and has a shorter learning curve, neuroendoscopic MVD is superior to microsurgical MVD in therapeutic effect, overall complications, and recurrence rate. Therefore, neuroendoscopic MVD may be an alternative to microsurgical MVD in the treatment of HFS in the Chinese population.

However, despite the promising clinical outcomes, neuroendoscopic MVD is surgically complex and necessitates a lengthier learning curve [5, 6]. In practice, we have encountered various challenges during the surgical process. Thus, in this study, we retrospectively analyzed the clinical data of 16 patients who underwent fully endoscopic MVD of the facial nerve through the Sigmoid sinus posterior approach for the first time in the Department of Neurosurgery at the First Affiliated Hospital of Bengbu Medical College, from August 2020 to July 2023. Through this retrospective analysis of prior cases, we aimed to consolidate surgical insights and assess clinical outcomes of fully endoscopic MVD in the treatment of HFS.

## Materials and methods

### Patient summary

Between August 2020 and July 2023, the Department of Neurosurgery at the First Affiliated Hospital of Bengbu Medical College treated 16 patients presenting with primary facial nerve spasms. Among them, 5 were male and 11 were female, with ages ranging from 30 to 66 years and an average age of 49 ± 10 years. All 16 patients exhibited unilateral facial spasms, with 11 on the right side and 5 on the left. The duration of the ailment varied from 3 to 84 months, with an average duration of 36.9 ± 25.7 months. Preoperative magnetic resonance imaging (MRI) scans with enhancement were conducted as standard protocol to rule out lesions in the cerebellopontine angle (CPA) area. Additionally, magnetic resonance tomographic angiography (MRTA) was conducted to confirm the presence or absence of blood vessels responsible for the spasms in the facial nerve root exit zone (REZ) area. A routine head computed tomography (CT) was performed on the first day after surgery. Inclusion criteria for the study comprised a preoperative imaging examination to exclude other intracranial lesions, absence of comorbidities and underlying diseases, and no prior surgical treatment for facial spasms. The study adhered to the principles outlined in the Declaration of Helsinki. Patients or their families were duly informed and provided their consent by signing the surgical informed consent form.

Preoperative grading was based on Cohen scale of HFS intensity. Postoperative efficacy evaluation was divided into immediate cure (spasm disappeared immediately after surgery), delayed cure (spasm disappeared gradually within 1 week to 6 months after surgery), recurrence (facial spasm disappeared after surgery and then recurred after a period of time), and no cure (facial spasm did not disappear after 6 months).

### MVD treatment

Under general anesthesia, neuroendoscopic MVD of the facial nerve was performed through the sigmoid sinus posterior approach, under vigilant neurophysiological monitoring.

**Fig. 1:**
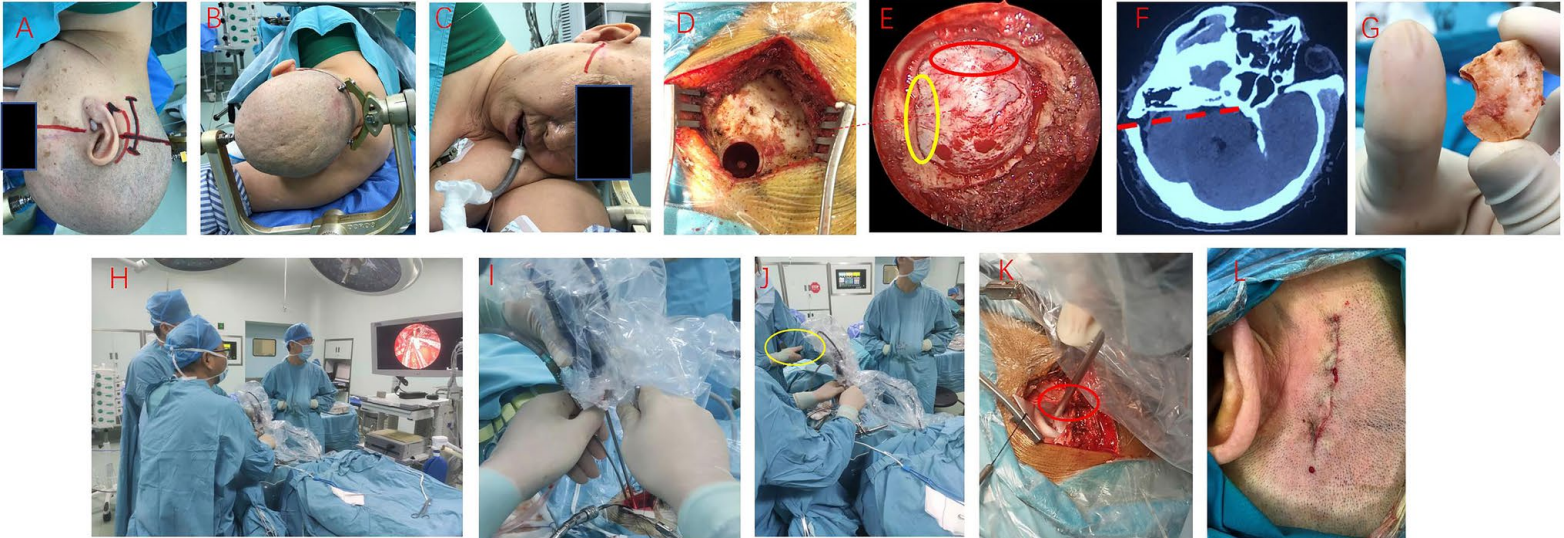
Procedure for fully endoscopic microvascular decompression of hemifacial spasms. **A** A curved incision behind the ear is depicted by the black line. **B** Position the patient in the lateral decubitus position with the mastoid tip at the highest point and secure the head with a three-pin headrest. **C** Slightly flex the head forward, maintaining a distance of two fingerbreadths between the mandible and the chest wall to avoid excessive flexion of the head. **D** Perform burr hole drilling. **E** The exposure range of the bone window is indicated, with the sigmoid sinus marked by a red circle and the transverse sinus by a yellow circle. **F** Ensure that the sigmoid sinus side of the bone window is fully exposed and level so that this edge aligns with the posterior edge of the petrous bone in a straight line, as indicated by the red dashed line. **G** The size of the bone flap should be similar to the size of the volar surface of the thumb. **H** The assistant holding the endoscope is positioned to the dominant left side, the endoscopic screen is placed opposite the surgeon, and the scrub nurse is positioned diagonally opposite the surgeon. **I** Two-person, three-handed “triangular three-point” microsurgical technique: the assistant holds the apex, while the surgeon uses both hands with the microscope positioned at the two base points for instrument manipulation. **J** One support point for the assistant holding the endoscope is the edge of the bone window at the sigmoid sinus side (yellow circle). **K** Another support point for the assistant holding the endoscope (red circle). **L** Intradermal suturing along the skin eversion line

**Fig. 2:**
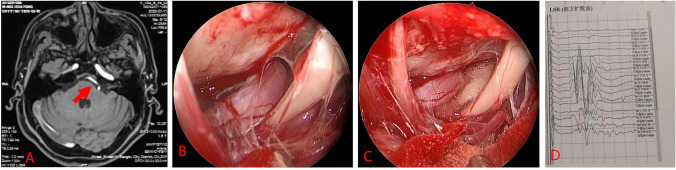
Case 1: male, 51 years old. **A** Preoperative magnetic resonance tomographic angiography (MRTA) indicates left vertebral artery compression of the facial nerve, with the red arrow pointing to the left vertebral artery. **B** Intraoperative observation shows the vertebral artery compressing the facial nerve. **C** Insertion of a spacer. **D** Laser speckle rheology (LSR) monitoring indicates the disappearance of abnormal waves after spacer insertion

**Fig. 3:**
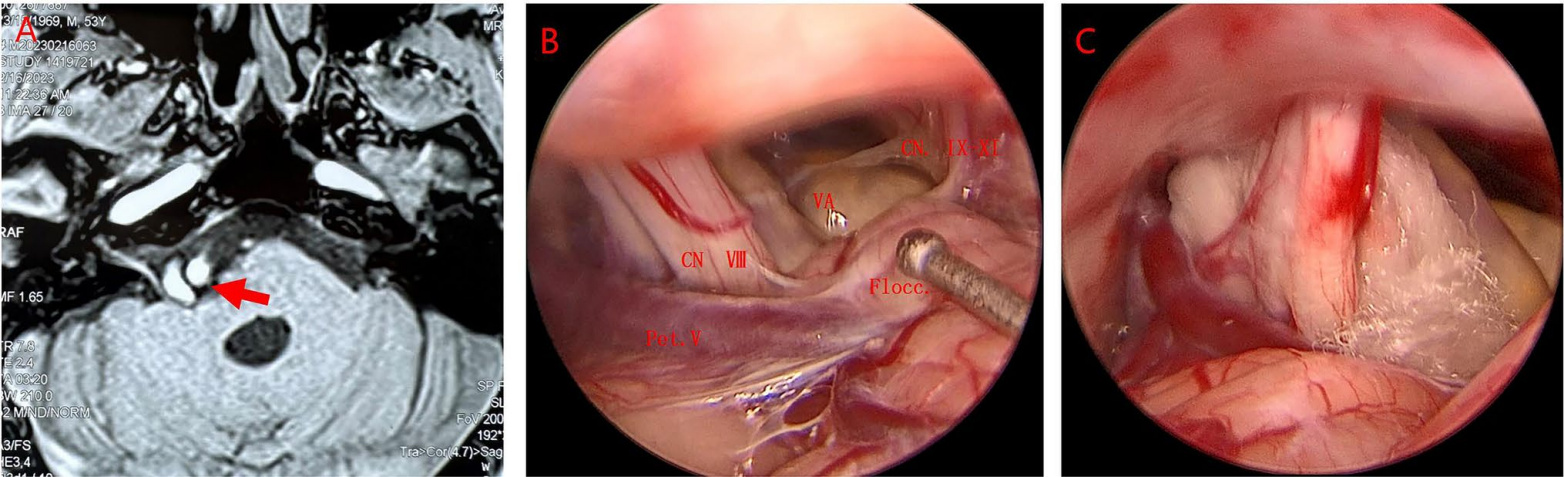
Case 2: male, 53 years old. **A** Preoperative magnetic resonance tomographic angiography (MRTA) suggests the right vertebral artery is exhibiting a climbing pattern compressing the facial nerve, with the red arrow pointing to the left vertebral artery. **B** Intraoperative observation reveals the right vertebral artery compressing the facial nerve. **C** Insertion of a spacer

**Fig. 4:**
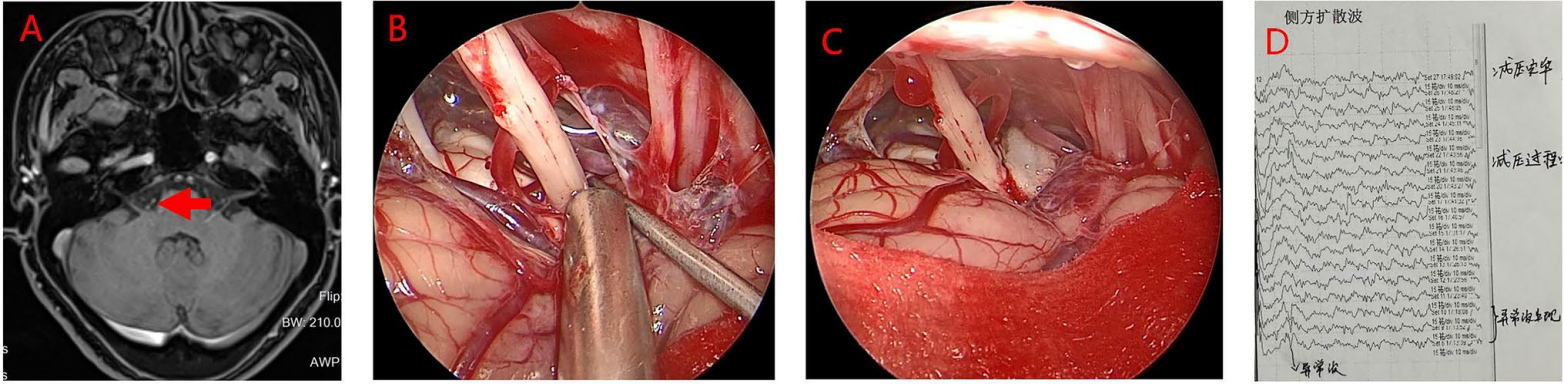
Case 3: female, 51 years old. **A** Preoperative magnetic resonance tomographic angiography (MRTA) suggests compression of the facial nerve root exit zone (REZ) by the right anterior inferior cerebellar artery, with the red arrow pointing to the right anterior inferior cerebellar artery. **B** Intraoperative observation shows the right anterior inferior cerebellar artery in a climbing pattern compressing the facial nerve. **C** Insertion of a spacer. **D** LSR indicates the disappearance of abnormal waves

The patient was positioned in a lateral prone stance, securing the head with a Mayfield head frame. The lower jaw was positioned to protrude at a width of two transverse fingers from the sternum, ensuring that the base of the mastoid was elevated to the highest point. An arcuate incision was carefully made in the postauricular region (Fig. 1a–c), allowing exposure of the asterion and the two-belly muscle groove. Subsequently, the asterion was carefully drilled (Fig. 1d). The bone was then milled open using a milling cutter, necessitating only one milling to fully expose the transverse and sigmoid sinuses, with particular emphasis on the sigmoid sinus aspect. After freeing the bone flap, the inner edge of the sigmoid sinus was navigated along the epidural space using a stripper, followed by further enlargement of the bone window with a milling cutter after detaching the sinus wall from the inner skull plate (Fig. 1e, f). The dura mater was routinely incised, releasing cerebrospinal fluid. Once the intracranial pressure subsided and the cerebellum retracted satisfactorily, the fully endoscopic (Karl Storz 30° endoscope) procedure commenced. The assistant held the endoscope while the surgeon conducted micro-operations using both hands (viewed by Karl Storz Ultra HD display) (Fig. 1h–k). Gradually, the facial nerve REZ area was accessed, and the offending blood vessels were carefully identified (as illustrated in typical cases shown in Figs. 2, 3, and 4). Following this, the pad was placed. Neurophysiological monitoring indicated the disappearance of the lateral spread response (LSR) of the facial nerve. The dura mater was sutured, the bone flap was repositioned and securely fastened with two peptide chains. Subcutaneous tissue was sutured, and the skin was closed using inverted sutures (Fig. 1I).

### Results

Preoperative HFS grading based on the Cohen scale showed that the cases in this study comprised 10 cases categorized as Cohen grade 3 and 6 cases categorized as Cohen grade 4. Using the postoperative efficacy evaluation criteria, there were 15 cases of immediate cure and 1 case of delayed cure.

In all 16 cases, offending blood vessels were successfully located during the operation and thoroughly padded. This included 12 instances of anterior inferior cerebellar artery (75%), 3 cases of vertebral artery (18.75%), and 1 case of posterior inferior cerebellar artery (6.25%).

LSR is an abnormal muscle response related to HFS observed during electromyography monitoring. Intraoperative LSR monitoring is crucial for the success of surgery, and appropriate anesthesia technique and electrode positioning are essential for accurate monitoring of LSR, while stimulation parameters should be carefully adjusted to avoid artifacts[7]. Disappearance of LSR during surgery correlates with short-term prognosis, but persistence of LSR does not necessarily indicate a poor long-term prognosis. LSR monitoring has positive and negative prognostic value, and its predictive power varies from study to study. Early LSR disappearance can occur before decompression and may predict a better clinical outcome. The application of LSR monitoring in HFS surgery requires further investigation. In this study, the intraoperative LSR disappeared completely in 15 cases and did not change in 1 case, but the symptoms of this patient disappeared after surgery.

Among all 16 cases in this study, there were two cases with postoperative complications (2/16, 12.5%). One case involved intracranial infection, which was successfully treated through lumbar cistern external drainage and anti-infection therapy. The other case experienced tinnitus, which was alleviated following oral treatment with mecobalamin and nimodipine.

## Discussion

By targeting the cause of HFS, MVD improves pain symptoms in patients by relieving nerve compression. MVD has a good effect in the treatment of HFS, reducing the oxidative stress reaction of surgical trauma, reducing the degree of pain in patients, and reducing the occurrence of postoperative complications and recurrence of HFS [8].

In this study, all 16 patients experienced unilateral onset of HFS, with a higher incidence on the right side (11 cases, 68.75%) than the left side, and a higher proportion of females (11 cases, 68.75%). A previous study reported that the incidence of HFS in Norway is 9.8 per 100,000 people, with Asians having a higher prevalence than whites, but the study lacked underlying population data to arrive at an accurate prevalence. In 1990, the total prevalence rate reported in the USA was 11 per 100,000, twice as high in women as in men, and most patients were 50 to 60 years old, which is consistent with our study [9, 10]. The primary cause of HFS is compression by implicated vessels, including the anterior inferior cerebellar artery, vertebral artery, posterior inferior cerebellar artery, and veins [11, 12]. Previous research [13] suggests that age alone is not a contraindication for surgery, nor is it a risk factor for MVD. MVD is an effective treatment for older and younger patients; the youngest participant in this study was 30 years old and the eldest was 66 years old.

MVD is the first-line treatment for HFS [3, 14–18]. Prior studies [19] indicate that the success rate of MVD in managing HFS can reach 95.37%, with a recurrence rate not surpassing 2.4%. Fully endoscopic MVD has considerable efficacy in treating HFS, boasting a 91.1% success rate in 39 cases reported by Feng et al. [17], and an 88.9% success rate in 54 cases documented by Zhu et al. [18]. In our cohort of 16 cases, the technique achieved a surgical success rate of 100%; however, the sample size was limited and warrants further validation.

The most frequent complications associated with MVD treatment for HFS are facial nerve paralysis and hearing loss, followed by instances of intracranial infection and wound-related issues, among others [20, 21]. Peng et al. [22] confirmed that fully endoscopic MVD surgery can reduce recurrence and complications, which is consistent with the results of this study. This may be because fully endoscopic MVD surgery has a better field of vision under the guidance of neuroendoscopy and can display the compression position more clearly through the high Telfon gasket, guiding the surgeon to rapidly and accurately relieve the compression. After surgery, warm saline can be injected under the direct vision of neuroendoscopy to imitate normal brain fluctuations and check whether the retraction of the offending blood vessel is effective, thereby reducing postoperative complications and recurrence. [4]. Neuroendoscopy can be used for close inspection through the space in the Regiones pedunculi cerebelli. Abnormal petrosus exist, and a better surgical field of view in HFS can be obtained via neuroendoscopy without bone removal or excessive stretching of the brain tissue around the operative area, thus reducing damage to blood vessels, nerves, and brain tissue in the operative area, and further reducing the possibility of postoperative complications and recurrence. In this study, there were two cases of postoperative complications (2/16, 12.5%). One case involved intracranial infection, which was successfully treated through lumbar cistern external drainage and anti-infection therapy. The other case experienced tinnitus, which was alleviated following oral treatment with mecobalamin and nimodipine.

Facial nerve MVD surgery primarily employs a microscope, a combination of a microscope and a neuroendoscope, or only a neuroendoscope. Microscopic MVD is the conventional approach [23]. However, owing to its limited field of view, the microscope cannot adequately visualize deeper structures, particularly the ventral side of the facial nerve REZ area. This makes it challenging for the surgeon to accurately identify all potential offending vessels that may be in contact with or compressing the facial nerve REZ area [17]. The primary cause of MVD decompression failure is oversight of offending vessels [24, 25], followed by insufficient decompression, Teflon adhesion, and improper utilization of decompression materials [15]. Some studies suggest that when multiple offending vessels are present in the facial nerve REZ area, they may be difficult to discern under a microscope [26]. The rapid advancement of endoscopic technology in recent years means the endoscope offers advantages such as a broad field of view, bright illumination, unobstructed visualization, and flexible maneuverability [27]. In comparison to microscopic MVD, the neuroendoscope excels in accurately identifying offending vessels [17, 27]. Some scholars have affirmed that fully endoscopic facial nerve MVD is safe and effective, although it presents challenges and has a steep learning curve [5, 6].

Fully endoscopic MVD treatment for HFS boasts numerous benefits. First, the superior visual field provided by the endoscope assists the surgeon in precisely identifying vessels and nerves within the surgical area, thereby averting damage to adjacent brain tissue, vessels, and nerves, and reducing postoperative complications [14]. Second, the endoscope aids in promptly evaluating the position of the Teflon pad and the effectiveness of decompression, enabling the surgeon to adapt the surgical approach based on intraoperative conditions. Finally, fully endoscopic MVD can curtail the length of the surgical incision, minimizing excessive craniotomy damage, avoiding undue arachnoid separation, and reducing traction on brain tissue and cranial nerves [28]. However, there are also certain drawbacks to fully endoscopic MVD. First, the neuroendoscope necessitates a specific operating space during surgery and can only offer two-dimensional images. Second, intraoperative bleeding or fogging may obscure the endoscope lens, potentially affecting image clarity and surgical continuity. Third, the learning curve for the endoscope is lengthier that that of the microscope [5]. Fourth, the endoscope requires an assistant or auxiliary arm for support. Lastly, the endoscope only provides a forward view and does not afford visualization of structures on both sides and behind the device. This could potentially lead to inadvertent damage to surrounding brain tissue, vessels, or nerves when adjusting the position and angle of the endoscope [29].

In early experience with fully endoscopic MVD surgery, in addition to the aforementioned issues, there were certain specificities that required attention. First, the Sigmoid sinus posterior approach to facial nerve MVD accesses the lateral cerebellum, which provides limited space for deep endoscopic operation. It is crucial to pre-release cerebrospinal fluid in the prepontine cistern. We find that using an endoscope for this task is challenging and may potentially harm the cerebellar cortex. Hence, we still rely on a microscope or the naked eye for this step. Once intracranial pressure drops and a gap is created, we transition to endoscopic operation. Second, improper positioning and inadequate exposure of the bone window, especially on the sigmoid sinus side, can lead to an excessive insertion angle for the endoscope. Challenges arise if the assistant is unable to hold the mirror steadily for an extended period, or if the auxiliary arm, while stable, cannot ensure continuous operation (Table 1).

**Table 1:** Baseline characteristics of patients

Case	Gender	Age	Hospitalization (days)	Course of disease (months)	Affected side	Responsible vessel	Complication
1	Male	30	17	24	Right	Anterior inferior cerebellar artery	/
2	Female	51	14	72	Right	Anterior inferior cerebellar artery	/
3	Female	42	14	72	Right	Anterior inferior cerebellar artery	/
4	Male	53	11	12	Right	Vertebral artery	/
5	Female	33	9	6	Right	Anterior inferior cerebellar artery	/
6	Female	54	20	6	Right	Anterior inferior cerebellar artery	/
7	Male	60	11	12	Right	Anterior inferior cerebellar artery	/
8	Female	59	18	36	Left	Anterior inferior cerebellar artery + vertebral artery	/
9	Female	55	14	60	Right	Vertebral artery	/
10	Female	50	16	72	Right	Anterior inferior cerebellar artery	/
11	Female	34	10	24	Left	Anterior inferior cerebellar artery	/
12	Female	52	14	36	Left	Posterior inferior cerebellar artery	/
13	Male	44	8	3	Left	Posterior inferior cerebellar artery + Vertebral artery	/
14	Female	66	12	60	Left	Anterior inferior cerebellar artery	/
15	Female	52	25	36	Right	Anterior inferior cerebellar artery	Intracranial infection
16	Male	50	17	60	Right	Anterior inferior cerebellar artery	/

For positioning, we employ the lateral decubitus posture, ensuring the mastoid is elevated. We recommend using a three-point head pin to secure the head, facilitating adjustments to the surgical field position by rotating the operating table during the procedure. The shoulder on the surgical side is pulled and fastened to the opposite side and the back, aiding in full exposure of the surgical area, and ensuring the assistant holds the mirror with minimal tilt. Some studies [30] have noted potential complications with three-point head pin fixation, such as changes in hemodynamics, skull fractures, and venous thromboembolism. Thus, it is essential to review the preoperative CT bone window position of the head when utilizing the three-point head pin. The incision can be either horizontal or arc-shaped, as long as it adequately exposes the asterisk, the mastoid groove, and the two abdominal muscle grooves. The sigmoid sinus side of the bone window should be as close to the mastoid direction as possible, with the bone edge smoothed to prevent endoscope obstruction or slippage upon entry. Preoperative review of CT axial positioning of the mastoid and MRI axial positioning is necessary to indicate skull and mastoid pneumatization at the Sigmoid sinus.

For endoscope handling, we have experimented with mechanical arms, pneumatic arms, electromagnetic arms, and other auxiliary supports. However, a common drawback in all these devices is their inability to ensure uninterrupted surgical operations. In instances where the endoscope lens is frequently obscured by blood or fog and requires periodic cleaning, or when continuous adjustments to the position and direction of the endoscope are necessary for exploring the facial nerve REZ area and placing the pad, mechanical arms prove time-consuming and laborious for adjustment and have been discontinued. Pneumatic arms and electromagnetic arms are viable options for surgeons without an assistant to hold the mirror. Some studies suggest that an assistant holding the mirror tends to be more stable and effective than an auxiliary arm [28]. We employ the “two-person three-hand” approach, wherein the assistant holds the endoscope while the surgeon operates with both hands. The assistant stands on the left side of the surgeon, using their left hand to support the mirror. To ensure mirror stability, we utilize two support points: one is the endoscope support point, located at the center of the sigmoid sinus edge of the bone window, and the other is the left arm support point, which is the surgeon’s right hand supporting the assistant’s left elbow joint. This is followed by the intraoperative “two-person three-hand” maneuver, adopting the “triangle three-point” configuration. Here, the assistant holds the endoscope at the bone window support point, forming the apex of the “triangle,” while the surgeon’s hands handle the instruments at the two base points of the “triangle.” This arrangement not only provides an ample field of view but also ensures sufficient operating space for the surgeon. Finally, we employ an angled mirror, with 30° being the most commonly used angle. In comparison to a 0° mirror, the primary advantage lies in the “butterfly effect” of the 30° mirror. This enables a 360° field of view without necessitating changes to the position of the endoscope. When assessing offending vessels and the placement of the pad under the endoscope, it is crucial to note that the facial nerve REZ area is situated beneath the facial auditory nerve and above the posterior group of cranial nerves. Offending vessels are frequently found on the ventral side of the CPA. The endoscope accesses the REZ area through two channels: one between the facial auditory nerve and the posterior group of cranial nerves, and the other beneath the CPA.

Intraoperative electrophysiological monitoring of the LSR of the facial nerve is vital during the procedure of fully endoscopic MVR [28–30], with some studies indicating that the LSR is a critical reference for assessing the short-term and long-term efficacy of MVD for facial muscle spasms. In cases utilizing LSR monitoring during surgery, patients exhibited notably improved postoperative outcomes compared with those that did not undergo monitoring of the LSR [32]. LSR demonstrates high accuracy in short-term follow-up. However, in long-term follow-up, as most patients experience relief from spasms irrespective of the LSR status, the accuracy diminishes. During the operation, thorough exploration of the facial nerve is imperative. If LSR persists after the operation, this indicates favorable long-term outcomes [31]. Nonetheless, LSR is not an absolute indicator. In this study, one case demonstrated clear identification of offending vessels during the operation and their complete isolation; however, despite there being no change in LSR during the operation, the patient’s facial muscle spasms were completely alleviated post-operation. The specific reason for these observations remains unclear, underscoring the continued importance of comprehensive facial nerve exploration in the procedure.

This study does have some limitations. First, the sample size is relatively small, which may impact the statistical robustness of the results. Second, the study was conducted in a single-center setting; thus, its findings may not be universally applicable to other contexts. There is a need for future comprehensive data analysis from the center. Third, the follow-up period is relatively short—only 2 months for the shortest duration—which may not fully and accurately reflect postoperative conditions and the long-term outcomes remain uncertain. Some studies [33] have indicated disparities in the long-term prognosis of patients with HFS undergoing MVD. The studies also highlight factors such as diabetes, the nature of the offending blood vessel during surgery, and facial nerve discoloration as potential influencers of long-term prognosis. Future studies with larger cohorts and extended follow-up durations are essential to validate the conclusions of this study.

Training is essential for the complex surgical procedure of fully endoscopic MVD, and the procedure should not be undertaken by novices. There are three main steps for training. The first is adaptive training of neuroendoscopy itself. Although neuroendoscopy can provide a good deep illumination field, it is a two-dimensional image, usually termed “fisheye effect.” There is no three-dimensional image of the microscope, and the blind area around the neuroendoscope means that structures around the endoscope cannot be observed. At present, endoscope lenses of 0°, 30°, 70°, and so on are commonly used. The 0° lens provides a similar field of view to the microscope; however, in clinical practice, we often use the 30° angle lens, which can provide a more satisfactory surgical field under a small space. Because neurosurgeons often cannot adapt to the sudden use of neuroendoscopy, neurosurgeons need to conduct adaptive training in a professional neuroendoscopy training unit before performing endoscopic surgery on patients. The second step in training is to practice the anatomy of CPA through the retrosigmoid endoscopic approach. The third step involves the problem of holding the endoscope and the cooperation between the surgeon and the assistant. The time required for the whole procedure is directly related to the ability of the trainees.

## Conclusion

In conclusion, fully endoscopic MVD of the facial nerve is a safe and effective procedure. It boasts several merits, including a clear visual field, minimal damage to brain tissue, and a high success rate. However, the procedure does present certain challenges, such as a steep learning curve (rigorous endoscopic training and extensive experience in MVD surgery are crucial) and the necessity for an assistant or auxiliary arm to support the endoscope. Despite these hurdles, with the continual advancements in endoscopic technology and surgical expertise, we anticipate that fully endoscopic MVD will emerge as a leading treatment for HFS in the future.

## Data Availability

The data generated in this study can be requested from the corresponding author.
